# Changes in self-reported and parent-reported health-related quality of life in overweight children and adolescents participating in an outpatient training: findings from a 12-month follow-up study

**DOI:** 10.1186/1477-7525-11-1

**Published:** 2013-01-02

**Authors:** Emily Finne, Thomas Reinehr, Anke Schaefer, Katrin Winkel, Petra Kolip

**Affiliations:** 1 Bielefeld University, School of Public Health, Universitätsstraße 25, D-33615, Bielefeld, Germany; 2 Department of Pediatric Endocrinology, Diabetes and Nutrition Medicine, Vestische Youth Hospital, University of Witten/Herdecke, Dr.-F.-Steiner-Str. 5, D-45711, Datteln, Germany

**Keywords:** Overweight, Health-related quality of life, Intervention, Weight reduction, Children and adolescents

## Abstract

**Background:**

Health-related quality of life (HRQoL) was found to improve in participants of weight management interventions. However, information on moderately overweight youth as well as on maintaining HRQoL improvements following treatment is sparse. We studied the HRQoL of 74 overweight, but not obese participants (32.4% male, mean age = 11.61 ± 1.70 SD) of a comprehensive and effective six-month outpatient training at four time-points up to 12 months after end of treatment.

**Methods:**

HRQoL was measured by self-report and proxy-report versions of the generic German KINDL-R, including six sub domains, and an obesity-specific additional module. Changes in original and z-standardized scores were analyzed by (2×4) doubly multivariate analysis of variance. This was done separately for self- and proxy-reported HRQoL, taking into account further socio-demographic background variables and social desirability. Additionally, correlations between changes in HRQoL scores and changes in zBMI were examined.

**Results:**

There were significant multivariate time effects for self-reported and proxy-reported HRQoL and a significant time-gender interaction in self-reports revealed (p < .05). Improvements in weight-specific HRQoL were evident during treatment (partial η^2^ = 0.14-0.19). Generic HRQoL further increased after end of treatment. The largest effects were found on the dimension self-esteem (partial η^2^ = 0.08-0.09 for proxy- and self-reported z-scores, respectively). Correlations with changes in weight were gender-specific, and weight reduction was only associated with HRQoL improvements in girls.

**Conclusions:**

Positive effects of outpatient training on generic and weight-specific HRQoL of moderately overweight (not obese) children and adolescents could be demonstrated. Improvements in HRQoL were not consistently bound to weight reduction. While changes in weight-specific HRQoL were more immediate, generic HRQoL further increased after treatment ended. An extended follow-up may therefore be needed to scrutinize HRQoL improvements due to weight management.

**Trial registration:**

clinicaltrials.gov NCT00422916

## Background

Overweight and obesity pose an increasing health problem in most industrialized countries [1,2]. The German Health Interview and Examination Survey for Children and Adolescents (KiGGS) showed that 8.7% of children and adolescents aged 3 to 17 years meet the national definition of overweight and additional 6.3% are obese [3]. Besides the long-term effects on morbidity and mortality [4,5], the immediate psychosocial consequences of excess weight like impaired quality of life and reduced self-esteem are of specific concern [6,7].

Health-related quality of life (HRQoL) relates to the self-perception of health and consists of ratings of well-being and functionality in important life areas, including physical functioning, bodily pain/symptoms, emotional well-being, self-esteem, social functioning and family relations [8,9]. While generic HRQoL allows comparison of these dimensions with healthy populations, disease-specific measures focus on impairments due to a specific health-condition and may therefore be more sensible to changes by means of treatment [10].

Research shows that in overweight or obese youth weight-specific as well as generic HRQoL is likely to be impaired [7,10-12], even when no objective disease markers can be observed [13]. This is to be expected in only modestly overweight children and adolescents, since manifest symptoms are rather unlikely at this age, but decreased self-esteem or psychological impacts may result from a negative body image and stigmatization of overweight youngsters [7,13,14].

Correspondingly, HRQoL was found to improve in participants of weight management interventions [11-13,15-20]. Improved well-being amongst other things may constitute a motivation for persisting behavioural changes that prevent weight re-gain after treatment. It was therefore suggested to include HRQoL as outcome measure in weight management programmes [7,13,17]. However, most studies so far have only focused on obese children and adolescents. Studies on moderately overweight children and adolescents remain to be done as well as studies with longer follow-up measures.

The aim of the present paper was to analyze changes in HRQoL among overweight children and adolescents participating in a six-month outpatient training programme for weight reduction with a follow-up period of 12 months and to relate changes in weight to changes in HRQoL. We expected a positive impact for weight loss on HRQoL.

## Methods

### Design and participants

The study was designed to be a randomized controlled trial (RCT) with a waiting list control group (participation in the intervention after six months without any intervention) to assess the effects of a six-month outpatient training on weight reduction and different secondary outcomes, including HRQoL. Details and results of the main study are described elsewhere [21,22].

Families were invited for participation in the study mainly by media (newspapers, radio) and paediatricians. To be included in the study, children had to be 8 to 16 years old, overweight, apparently healthy and not be taking any medication. Overweight was defined as a BMI ≥90^th^ percentile and ≤97^th^ percentile, according to German percentiles [23]. Obese children were excluded from the sample. For the present analysis, participants of the RCT and participants of the treatment who did not take part in the randomized part of the study were investigated. All eligible children and adolescents enrolled for the treatment from January 2007 to mid-July 2009 (follow-up 2 until end of 2010) constituted the master sample (including n = 66 participants of the RCT, n = 19 participants of a pilot study, and n = 33 enrolled after end of the recruitment period for the RCT).

Participants differed in the time period that elapsed between study enrolment and start of intervention. Children of the pilot study and those from the RCT control group (n = 32) had to wait six months before entering the treatment, while children of the RCT intervention group (n = 34) and those enrolled after end of recruitment for the RCT were assigned to the next available training course. Participants with longer waiting periods did not differ in any of the compiled characteristics from those who started the intervention directly at beginning of the treatment, and participants of the RCT did not differ from those not randomly allocated. Furthermore, length of the waiting period before intervention did not affect the results described. The groups were therefore combined for the presented analysis. The final sample, after exclusion of 30 dropouts before beginning of training, 10 cases ineligible at start of training (n = 8 obese and n = 2 normal weight), and 4 cases with missing questionnaires at second follow-up, consisted of 74 children.

HRQoL and weight status of all participants were measured at beginning (pre-treatment) and end (post) of the six-month training, as well as six (follow-up 1) and twelve months (follow-up 2) after end of intervention. In order to follow an intention-to-treat approach, missing values due to drop-out during intervention (n = 2) or lost to follow-up after end of intervention (n = 1) were set back to baseline values and included in the analyses. Excluded cases were compared with the analyzed sample and no significant differences were found regarding age, weight status, gender, foreign background, socio-economic or family status, social desirability or baseline HRQoL scores (p > .05).

### Ethical approval

Written informed consent was obtained from all children and their parents prior to study start. The study was approved by the local ethics committee of the University of Bremen.

### Intervention

The six-month outpatient training “Obeldicks light” was offered in two cities in north-west Germany. The intervention has been described in detail [24]. Briefly, the intervention was based on participation in weekly physical activity sessions (1.5 hours per session over 6 months, including ball games, jogging, dancing -girls, and wrestling -boys), nutrition education (based on the ‘Optimized Mixed Diet’ [25]), and behaviour counselling (67 hours of intervention in total). Interventions were performed in group sessions and individual counselling for the child and his/her family.

Previously published analyses showed that the intervention was successful in significantly reducing several parameters such as body weight, body fat percentage, waist circumference, and blood pressure as compared to the control group at the end of treatment. 94% of children reduced their body mass index z-score (zBMI) during the training and 24% reached normal weight [21]. Follow-up results showed that weight changes remained stable at least until one year after end of treatment [22]. Preliminary analyses revealed HRQoL changes in favour of the intervention group during treatment. These were, however, only significant for weight-specific HRQoL [26].

### Measures

#### HRQoL

Health-related quality of life was measured by German age-specific self-report versions and parent-proxy versions of the KINDL-R questionnaire [27]. KINDL-R is a generic HRQoL measure that distinguishes six dimensions with reference to the last week: physical (e.g. “I felt ill”) and emotional well-being (e.g. “I had fun and laughed a lot”), self-esteem (e.g. “I was proud of myself”), family (e.g. “I got on well with my parents”), friends (e.g. “I got along well with my friends”), and school (e.g. “Doing the schoolwork was easy”). In the parent-versions analogous items were answered by proxy. Each dimension is measured by 4 items and transformed to a range from 0 (low) to 100 (high). A total score for overall HRQoL from 0 to 100 can also be computed. Parents and adolescents also filled in the 12-item disease-specific obesity module of KINDL-R. To reduce the burden on younger children, for children aged 8-11 years the questionnaire was shortened, and therefore did not include the obesity module.

The KINDL-R was chosen because of its sensitivity to change [8,27] and because the disease-specific module as well as German norms are available [28]. It showed acceptable reliability and validity in different applications [12,28,29]. Cronbach’s alphas in this study were α > 0.80 for the self-reported and parent-reported total scores as well as weight-specific HRQoL scores. Cronbach’s alphas for the generic HRQoL subscales varied from α = 0.54 to 0.80 with the lowest reliability for the friends subscales and values α < 0.70 for self- and proxy-reported self-esteem and school as well as parent-reported emotional well-being and self-reported physical well-being.

HRQoL measures were z-standardized using German norms from a recent representative sample [28] to allow for easier interpretation of the scores relative to the population and to compensate for age-typical changes in HRQoL. Since population norms for the child self-report version of the KINDL-R are only available from 11 years upwards, for the younger children norms of an 8-12 year-old sample from the KINDL-R manual [27] were applied. Because HRQoL was measured every six months over a period of 1.5 years, some children shifted reference category between two measurement points, which may have resulted in discontinuities or leaps in the scores. Moreover, there are no norms available for the disease-specific obesity module. To also allow interpretation of absolute changes in HRQoL, KINDL-R original 0-100 scores were, therefore, analyzed in separate models.

#### Anthropometry

To calculate children’s BMI, height was measured to the nearest centimetre using a rigid stadiometer. Weight was measured in underwear to the nearest 0.1 kg using a calibrated balance scale. The degree of overweight was quantified using Cole’s least mean square method expressing BMI as a standard deviation score (zBMI) [30] using reference data for German children [23]. HRQoL, weight and height were measured every six months including one measurement before start of treatment (pre), one measurement at the end (post), six months later (follow-up 1) and again twelve months later (follow-up 2).

#### Background variables

Background variables such as socio-economic status (SES), ethnicity and family status were assessed by parent questionnaires [31,32]. SES was based on parents’ education, occupational status, and household income. A foreign background was inferred, if one or both parents’ country of birth was not Germany. Social desirability was assessed by questionnaire in parents [33] and youth [34]. Background variables were measured once at start of the study.

### Statistical analyses

#### Data screening and missing values

Data were screened for missing values and outliers. As missing values on single occasions and subscales were common and Little’s MCAR test indicated missingness at random, individual missing values were estimated by a formula based on the individual mean throughout measurements and the group mean at the measurement in question [35]. In general, the proportion of missing values was below 5%, except for some parent proxy-reported values related to school and friends. The proportion of missing values was highest for ‘school’ reported by parents at follow-up 1 and 2 (10.8% each).

Not all scales showed normal distribution. Since distributions differed between subscales and time points, no uniform normalizing transformation was possible. However, sample size was large enough to assume robustness of MANOVAs [35].

HRQoL data were tested for univariate and multivariate outliers by inspection of boxplots and Cook’s distance. There was one case with a high Cook’s distance (1.85). However, as MANOVA results were very similar with and without this case, we included it unchanged in the analyses.

#### Main analyses

Descriptive statistics were computed for boys and girls and the total sample and compared using independent t-tests for continuous measures and chi-square-tests for categorical variables. For the main analyses doubly multivariate analyses of (co-)variance were computed separately for child and parent-proxy HRQoL original and z-scores to compare the four measurements from pre to follow-up 2 using GLM procedures of IBM SPSS Statistics 20.0 (IBM Corporation, Somers, NY, USA). In subsequent univariate analyses successive HRQoL scores were contrasted with pre-treatment scores.

Given that weight-specific HRQoL and weight complaints were only reported by adolescents (n = 25), these two variables were examined univariately only and not included in the multivariate child models. Aside from that, there were no z-scores for weight-specific HRQoL and weight complaints, so MANOVAs on z-scores were constrained to generic HRQoL.

In all models gender and SES were included as potential between-subjects factors, testing the main effects and interactions with time. Because of resulting small group sizes, these factors were only included in the final models, if there was a significant interaction with time. Furthermore, duration of the waiting period between study enrolment and start of the training, baseline age, zBMI at baseline, and social desirability were tested as potential covariates and included in the final model, when there was a significant interaction with time.

Spearman’s rank order correlations between changes in zBMI over the course of the treatment (short-term from pre to post) as well as from start of training until the 12-month follow-up (long-term from pre to follow-up 2) and short- and long-term changes in HRQoL scores were analyzed. Since we expected weight reduction to correlate with HRQoL improvements, significances of the correlations are given for one-tailed tests.

Unless otherwise specified, p-values ≤ 0.05 were considered significant.

Power analysis revealed that with a sample size of 74 and correlations between repeated measures of r = 0.5, an effect of medium size would be detected with a power > 0.95, and the power for univariate contrasts exceeded 0.80 after adjusting alpha-level for multiple tests. For self-reported weight-specific HRQoL in adolescents (n = 25), however, a power > 0.80 was given for large effects only [36].

## Results

### Sample description

Table 1 shows descriptive statistics of the sample. Nearly two thirds of participants were girls and nearly two thirds were recruited from the RCT. One third were adolescents (12 years or older). The sample showed a SES-distribution similar to that of the German population [31] but the proportion of children of foreign background was significantly lower in our sample than in the German population (13.5% vs. 25.4%) [32]. Male participants were slightly more overweight than females.

**Table 1:** Sample description

	**Total**	**Girls**	**Boys**	**Girls vs. Boys**^**1**^
N	74	50	24	<.01
age at start of training (M; SD)	11.61; 1.70	11.46; 1.74	11.86; 1.61	ns
% adolescents	33.8	32.0	37.5	ns.
% RCT sample	64.9	62.0	70.8	ns
% proxy-report by mother	81.1	84.0	75.0	ns
**SES:**				ns
% low	28.2	29.2	26.1	
% medium	47.9	43.8	56.5	
% high	23.9	27.1	17.4	
% single parent	17.6	18.0	16.7	ns
% foreign background	13.5	14.0	12.5	ns
BMI (M; SD)	23.67; 1.48	23.44; 1.34	24.14; 1.68	<.10
zBMI (M; SD)	1.64; 0.18	1.61; 0.18	1.70; 0.16	<.05
**Social desirability (M; SD)**				
Parents honesty (z-score)^2^	−0.88; 0.86^a^	−0.92; 0.81	−0.79; 0.98	ns
Children (T-score)^3^	56.82; 8.90^b^	55.69; 9.20	59.19; 7.87	ns

^1^ p-value for the comparison between girls and boys.

^2^ lower scores for ‘honesty’ imply higher social desirability.

^3^ higher scores for higher social desirability.

ns = not significant (p > .10).

a significantly lower than expected population mean (0), p ≤ .001.

b significantly higher than expected population mean (50), p ≤ .001.

Table 2 lists BMI and HRQoL scores over the course of the study. As the z-scores show, HRQoL in this moderately overweight sample was slightly impaired for most of the subscales before treatment. Deviation from age- and gender-specific norms, however, was mainly significant for parent proxy-reported HRQoL. The only dimension significantly impaired according to self-reported values was social functioning in terms of friends.

**Table 2:** Body mass and HRQoL scores over the course of the study, M (SD)

		**Start of training (pre)**	**End of training (post)**	**Follow-up 6 months (follow-up 1)**	**Follow-up 12 months (follow-up 2)**	**Partial η**^**2**^**for univariate time effect**
BMI		23.67 (1.48)	23.01 (1.58)	23.49 (1.84)	23.94 (2.09)	0.141
zBMI		1.64 (0.18)	1.38 (0.29)***	1.41 (0.36)***	1.41 (0.43)***	0.251
**Parent proxy-report**						
total HRQoL	raw	73.18 (10.51)	75.67 (9.46)	74.14 (10.91)	76.53 (8.40)**	0.053
	z-score	−0.44 (1.11)^**c**^	−0.10 (1.02)*	−0.17 (1.12)	0.11 (0.85)***	0.096
physical	raw	74.79 (16.01)	76.78 (15.15)	75.86 (18.06)	77.32 (16.67)	0.011
	z-score	−0.20 (0.99)	0.01 (0.96)	0.01 (1.15)	0.15 (1.00)**	0.031
emotional	raw	76.39 (13.65)	79.14 (11.50)	77.82 (14.47)	79.24 (15.10)*	0.028
	z-score	−0.38 (1.10)^**b**^	−0.11 (0.91)*	−0.18 (1.13)	−0.05 (1.14)**	0.048
self-esteem	raw	64.70 (12.83)	70.02 (12.84)B**	66.51 (15.36)	69.81 (12.50)**	0.062
	z-score	−0.37 (0.95)^**c**^	0.07 (0.99)B***	−0.15 (1.14)	0.12 (0.89)B***	0.081
friends	raw	72.54 (13.01)	73.02 (14.18)	72.90 (17.87)	76.99 (13.24)**	0.047
	z-score	−0.39 (0.98)^**c**^	−0.34 (1.06)	−0.33 (1.32)	−0.03 (0.99)B**	0.052
family	raw	75.27 (14.83)	77.21 (13.06)	77.32 (14.85)	77.32 (13.64)	0.011
	z-score	−0.22 (1.10)	−0.03 (0.91)	0.01 (1.02)	0.03 (0.93)*	0.028
school	raw	75.22 (15.92)	77.90 (13.10)	74.70 (16.01)	78.08 (12.77)*	0.031
	z-score	−0.29 (1.10)^**a**^	0.00 (0.95)*	−0.11 (1.06)*	0.21 (0.86)B***	0.098
weight-specific		71.34 (14.99)	78.74 (13.79)B***	78.57 (14.10)B***	79.78 (11.44)B***	0.136
weight complaints		1.91 (0.90)	1.51 (0.63)B***	1.58 (0.65)B***	1.49 (0.65)B***	0.125
**Child self-report**						
total HRQoL	raw	75.51 (11.37)	77.78 (10.25)	76.78 (11.90)	78.80 (9.95)**	0.036
	z-score	0.00 (1.27)	0.30 (1.11)*	0.24 (1.28)	0.51 (0.99)***	0.067
physical	raw	73.89 (15.90)	76.50 (15.24)*	75.73 (17.01)	76.88 (16.08)	0.020
	z-score	0.04 (1.09)	0.24 (0.97)*	0.21 (1.11)	0.35 (1.00)*	0.030
emotional	raw	82.60 (14.60)	82.14 (12.13)	82.62 (15.14)	85.45 (11.79)	0.005
	z-score	−0.03 (1.34)	−0.04 (1.07)	−0.01 (1.41)	0.28 (0.96)	0.007
self-esteem	raw	65.41 (16.97)	67.82 (15.11)	68.49 (20.17)	72.69 (15.54)**	0.043
	z-score	0.20 (0.96)	0.45 (0.91)	0.58 (1.13)*	0.83 (0.89)B***	0.093
friends	raw	75.94 (15.07)	79.60 (12.79)	77.77 (16.68)	80.17 (14.05)	0.016
	z-score	−0.24 (1.12)^**a**^	0.06 (0.93)	−0.10 (1.24)	0.11 (1.02)	0.021
family	raw	81.85 (16.30)	85.26 (14.29)	82.53 (16.03)	82.98 (15.56)	0.014
	z-score	−0.16 (1.23)	0.10 (1.05)	−0.08 (1.15)	0.01 (0.98)	0.014
school	raw	74.29 (19.11)	76.13 (17.64)	72.43 (18.84)	74.20 (15.93)§	0.020
	z-score	0.18 (1.39)	0.36 (1.17)	0.12 (1.20)	0.30 (0.92)§	0.027
weight-specific (n = 25)		75.36 (13.77)	83.59 (13.61)B**	82.69 (14.51)B**	82.30 (14.17)**	0.187
weight complaints (n = 25)		2.11 (0.91)	1.66 (0.83)B**	1.69 (0.85)B**	1.61 (0.78)**	0.194

a: p ≤ .05, b: p ≤ .01, c: p ≤ .001 compared to norm population.

* p ≤ .05, ** p ≤ .01, *** p ≤ .001 for simple contrast against pre score (unadjusted);

**B:** p ≤ .05 after Bonferroni correction for multiple tests; **B** ≤ .0021 for proxy-reported original scores, **B** ≤ .0028 for proxy-reported z-scores and self-reported original scores, **B** ≤ .0083 for self-reported weight-specific scores.

§ significant time-gender interaction.

BMI z-scores (zBMI) are based on Cole’s LMS method.

All HRQoL original scores range from 0 to 100, except for weight complaints that vary from 1 (never/not at all) to 5 (always/strong); z-scores are the deviance from the age- and gender-specific population mean in terms of SD units.

Effect size η^2^ is classified as 0.01 = small, 0.06 = medium, 0.14 = large effect.

Overweight according to zBMI was significantly reduced during treatment and remained relatively stable until follow-up 2 (see also [22]).

### Follow-up results

#### Parent proxy-reports

Neither age, length of the waiting period, baseline zBMI, nor social desirability as covariates showed significant main effects or interactions with time. There was also no significant main effect or interaction of SES or gender. For the raw scores there was a significant multivariate effect of time on HRQoL (see Table 3). HRQoL scores on most subscales increased during treatment, then slightly declined followed by a second increase between the first and second follow-up. Simple contrasts against pre-treatment scores were only significant for self-esteem post-treatment and weight-specific HRQoL when adjusted for multiple tests (see Table 2). Analogously, effect sizes for the univariate time effects on the generic subscales were low to moderate, while the effects on weight-specific HRQoL and weight complaints were large.

**Table 3:** Results of doubly multivariate MANOVAs on HRQoL scores over time

	**Pillai’s trace/ Wilks’ λ**	**F**	**df 1**	**df 2**	**p-value**	**partial η**^**2**^
**Parent proxy-report raw scores**						
time	0.463/ 0.537	1.80	24	50	<.05	0.463
**Parent proxy-report z-scores**						
time	0.409/ 0.591	2.16	18	56	<.05	0.409
**Children self-report raw scores**						
gender	0.033/ 0.967	0.38	6	67	ns	0.033
time	0.357/ 0.643	1.70	18	55	<.10	0.357
time × gender	0.403/ 0.597	2.06	18	55	<.05	0.403
**Children self-report z-scores**						
gender	0.068/ 0.932	0.82	6	67	ns	0.068
time	0.432/ 0.568	2.32	18	55	<.01	0.432
time × gender	0.367/ 0.633	1.77	18	55	<.10	0.367

ns = not significant (p > .10).

Dependent variables for parents’ raw scores were the six generic HRQoL scales, weight-specific HRQoL and weight complaints measured at four time points: pre- and post-treatment, at follow-up 1 and follow-up 2; for parents’ and children’s z-scores as well as for children’s raw scores dependent variables were the six generic HRQoL subscales at the four time points.

**Figure 1:**
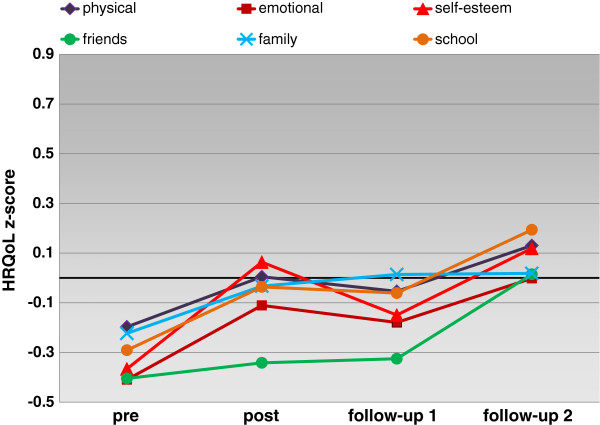
Profile of HRQoL z-scores (estimated marginal means) over time from parents’ proxy-reports.

For the z-scores the time effect was likewise significant (Table 3). Univariate simple contrasts were significant for self-esteem, friends, and school after adjustment for multiple tests. The profile plot of the z-scores (Figure 1) also shows that HRQoL scores still increased beyond end of treatment after levelling off. Not until 12 months after end of treatment did the scores consistently reach the population mean (z = 0). In general, increases in generic HRQoL were more visible with the z-scores than the original scores with slightly higher but still low to moderate effect sizes.

#### Child self-reports

For children’s self-reported raw scores we found a significant effect for social desirability, where children with higher scores had significantly higher HRQoL scores, but there was no interaction with time, so social desirability was not included in the final model. All other tested covariates and SES showed neither main effects nor interactions with time. The main effec of time only approached significance (p < .10), but there was a significant time-gender interaction in the multivariate test (Table 3). Univariate contrasts did only reach significance for weight-specific HRQoL after Bonferroni adjustment (Table 2). Concerning the time-gender interaction only one unadjusted contrast was significant for school at follow-up 2 (partial η^2^ = 0.10).

In terms of the z-scores, results for the covariates resembled those of the raw scores. There was a significant time effect, as well as a nearly significant time-gender interaction. Univariate contrasts showed that only self-esteem at follow-up 2 remained significant after Bonferroni adjustment. The unadjusted interaction contrast for gender × time was p < .05 for school at follow-up 2 (partial η^2^ = 0.07).

**Figure 2:**
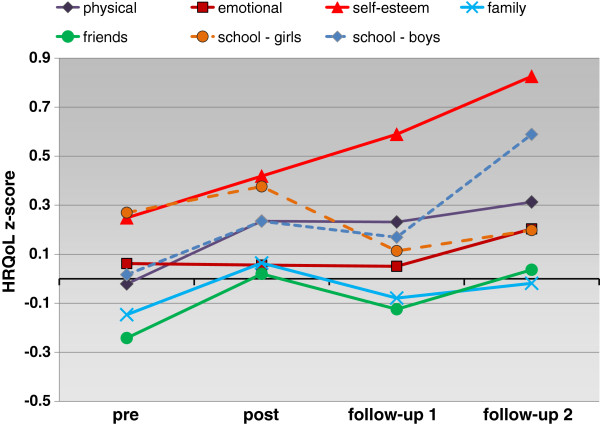
Profile of HRQoL z-scores (estimated marginal means) over time from children’s self-reports.

As the profile plot (Figure 2) shows, the most marked increase was for the subscale self-esteem. Physical well-being increased during treatment and remained relatively stable afterwards. Z-scores for school increased in boys but slightly decreased in girls. Only the subscales friends and family were below average at beginning of the intervention, and for both scales z-scores in tendency sloped upwards, reaching the population mean after training and at follow-up 2.

#### Weight-specific HRQoL

**Figure 3:**
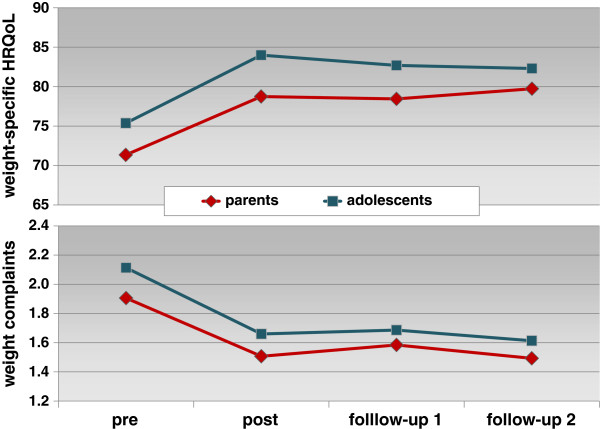
**Estimated marginal means of weight-specific HRQoL and weight complaints (parents’proxy-reports and adolescents’ self-reports).** Weight-specific HRQoL scores on a scale from 0 (lowest HRQoL) to 100 (highest HRQoL). Weight complaints were measured on a scale from 1 (never/not at all) to 5 (always/strong).

Nearly all univariate simple contrasts for the KINDL obesity module against baseline scores were significant. The profile plots (Figure 3) endorse a marked increase in weight-specific HRQoL and decrease in weight complaints during treatment and relative stable scores for all occasions till follow-up 2, 12 months after end of intervention.

### Correlations of changes in zBMI with changes in HRQoL

Change scores for HRQoL and zBMI were computed for short-term (post minus pre) and long-term (follow-up 2 minus pre) changes and Spearman’s rank order correlations between HRQoL and zBMI changes were computed for the entire sample as well as for girls and boys separately (see Table 4).

**Table 4:** Spearman’s rank order correlations between short-term and long-term changes in zBMI and changes in HRQoL for the entire sample (n = 74) and for girls (n = 50) and boys (n = 24) separately

		**Short-term zBMI change**		**Long-term zBMI change**		**Short-term zBMI change**				**Long-term zBMI change**			
**HRQoL changes:**		self-report	proxy-report	self-report	proxy-report	self-report		proxy-report		self-report		proxy-report	
		total	total	total	total	♀	♂	♀	♂	♀	♂	♀	♂
**total HRQoL**	short-term	.008	-.024	-.143	-.080	-.088	.131	.001	-.031	-.255*	-.006	-.196	.187
	long-term	.127	.189	-.006	-.056	.107	.189	.175	.187	-.170	.392*	-.191	.201
**physical**	short-term	-.218*	-.108	-.094	-.046	-.357**	.100	-.251*	.266	-.153	-.003	-.219	.395*
	long-term	.003	.087	-.118	-.101	.010	.054	.043	.110	-.170	.054	-.277*	.358*
**emotional**	short-term	-.002	-.093	-.150	-.021	-.080	.197	-.059	-.157	-.309*	.172	-.059	.059
	long-term	.112	.066	.038	-.151	.109	.124	.117	-.024	-.132	.467*	-.181	-.035
**self-esteem**	short-term	.009	-.002	-.226*	-.070	-.067	.236	-.044	.123	-.300*	-.078	-.166	.192
	long-term	.162	.142	-.017	-.140	.222	.009	.066	.306	-.011	-.045	-.208	-.018
**family**	short-term	.021	.017	-.068	.002	-.003	.057	.006	.094	-.039	-.160	-.056	.146
	long-term	.169	.119	.166	.024	.108	.321	.113	.154	.069	.368*	-.046	.168
**friends**	short-term	.179	-.140	.165	-.069	.155	.242	-.151	-.111	.047	.445*	-.156	.092
	long-term	.254*	.187	.058	.013	.190	.408*	.183	.166	-.123	.485**	-.055	.212
**school**	short-term	-.098	.073	-.243*	-.016	-.176	.164	-.025	.329	-.383**	.039	-.116	.182
	long-term	-.237*	.041	-.232*	-.117	-.237*	-.086	.167	-.041	-.380**	.124	-.115	.044
**weight-specific HRQoL**	short-term	-.176	-.218*	-.072	-.088	-.323	.218	-.234	-.232	-.281	.250	-.296*	.305
	long-term	.164	-.010	-.363*	-.135	.137	.127	-.040	.080	-.377	-.261	-.360**	.290
**weight complaints**	short-term	.417*	.300**	.120	.142	.568*	.111	.322*	.275	.496*	-.322	.253*	-.062
	long-term	.165	.061	.495**	.134	.406	-.191	.051	.096	.710**	.242	.311*	-.324

* p < .05, ** p < .01, ** p < .001 (one-tailed); ♀: girls; ♂: boys;

Short-term change = from pre- to post-treatment (pre minus post);

Long-term change = from pre-treatment to follow-up 2 (follow-up 2 minus pre);

Except for weight complaints, negative correlations imply that reductions in zBMI (negative change score) were associated with improvements in HRQoL (positive change score).

In general, correlations with generic HRQoL change scores were very similar for raw scores and z-scores, so only results for z-scores are given.

Results for weight-specific HRQoL revealed that weight reduction during treatment was significantly associated with improved parent-reported weight-specific HRQoL as well as with reduced self- and parent-reported weight complaints during the same timeframe. Long-term weight reduction was significantly associated with improvements in long-term self-reported but not parent-reported weight-specific HRQoL (including reduced self-reported weight complaints). Inspection of gender-specific correlations showed, however, that the described patterns were primarily true for girls.

In terms of generic HRQoL, short-term weight reduction was significantly correlated with improved physical well-being during treatment and long-term improvements in school-related HRQoL (self-reported). Self-reported short- and long-term improvements in school-functioning as well as short-term improvements in self-esteem were significantly associated with long-term weight reduction. All these relations, however, again were only present in girls. Girls’ self-reported changes in emotional well-being during treatment also correlated significantly with long-term weight changes.

Correlations contrary to the expected direction (namely that a reduction in zBMI would be associated with improved HRQoL) were found on some HRQoL subscales in boys, primarily for self-reported HRQoL. Some parent-reported HRQoL scores also declined while zBMI was reduced during treatment, revealing associations opposite to those in girls, but these correlations did not reach significance.

Significant associations were found between boys’ short-term changes in social well-being (friends) and weight complaints and long-term changes in zBMI, which indicate that improved well-being was rather associated with a weight gain or that reduced well-being in these domains preceded long-term weight reduction. Long-term weight reduction was furthermore significantly associated with reduced self-reported HRQoL total score, emotional, familial and social well-being as well as parent-reported physical well-being in the long run. Associations between boys’ weight reduction and decreased parent-reported weight-specific HRQoL showed the same trend, although not statistically significant.

The magnitude of correlations was low to medium in general, except for self-reported HRQoL in girls.

## Discussion

The main aim of the present study was to analyze changes in HRQoL during and after participation in an outpatient training programme for weight reduction in overweight children and adolescents. Furthermore, changes in HRQoL were related to weight changes.

Results from children’s self-report and parent proxy-report showed significant improvements in several HRQoL dimensions. All subscales reached or outreached population means at follow-up 2. The largest increases were found for weight-specific HRQoL during treatment. By contrast, subscales of generic HRQoL often continued to increase after end of treatment and were mostly not significantly higher than pre-treatment until follow-up 2 with the exception of parent-reported self-esteem. In general, parents reported more marked changes and changes were more obvious with age- and gender-specific norm scores than with raw scores. Correlations in the expected direction, namely associations of weight reduction with HRQoL improvements, were found primarily in girls.

Our results on HRQoL-improvements in participants of a successful weight management intervention are in line with the results of other studies that analyzed mainly obese children and adolescents [7,10-12,15,16,18,20]. However, unlike former studies we focused on moderately overweight youth and followed them over multiple measurements until 12 months after end of training. Short-term improvements in our overweight sample turned out to be smaller than those reported in other studies that were conducted among obese youth.

Compared to age- and gender-specific population norms [28], parent proxy-reports showed significantly impaired HRQoL pre-treatment scores in most subscales. There is no cut-off for clinically relevant HRQoL impairments or changes for the KINDL-R. However, compared to other studies on obese children where scores from 1 SD below the mean of healthy norms were regarded as impaired [37], baseline scores were only slightly reduced in our participants. Compared to a study that used the KINDL-R in mainly obese participants of an outpatient treatment [11], we found slightly higher self-report values for the total, emotional and the school score, very similar values for physical well-being, friends, and family, and notably higher values for self-esteem and weight-specific HRQoL. Weight-specific HRQoL was higher than in German overweight and obese youth of the same age-range seeking outpatient treatment in a recent multicenter study [38].

Concerning changes in HRQoL dimensions, we found the largest increases in weight-specific HRQoL during treatment. Because disease-specific instruments should be more sensitive to changes during treatment [39], this result was expected. It is in line with previous reports that also found clear improvements of this dimension from pre- to post-treatment in mainly obese youth [11,12,15,16,20]. Yet the revealed large effects outperform improvements of moderate effect sizes that were reported in most former studies, even if impairments in weight-specific HRQoL in our sample were rather less than in obese samples. However, as our results show, improvements were not constrained to weight-specific HRQoL but also affected on generic HRQoL.

In terms of generic HRQoL we found the most notable increases in self-reported as well as parent-reported self-esteem equivalent to a moderate effect-size, although not all contrasts reached significance. While self-esteem is not included in most HRQoL-instruments and was therefore not covered by all studies, some studies also demonstrated significant increases [12,16,20,40] while others found non-significant but similar absolute increases [11]. Along with the results of reviews on self-esteem in paediatric overweight [7,41] it can be concluded that weight management programmes positively impact on self-esteem in overweight and obese youth and that these improvements remain relatively stable over time. A striking result not reported by previous research was the high initial self-reported self-esteem in our sample, which further increased over time. It may be that youth with high self-esteem feel more confident to participate in a weight management intervention.

With respect to other HRQoL dimensions the literature reveals inconsistent results which may be due to different instruments, self-report versus parent proxy-report versions and differences between samples and treatments. However, studies with obese participants found significant HRQoL increases on at least some HRQoL subscales during treatment [10]. Griffiths et al. [7] in their review confirmed an improvement for most HRQoL dimensions except for school functioning (inconsistent results) and family (rarely studied). From our results we can confirm increases during treatment, but in our sample improvements were more pronounced in the long run and looking at parent-reported and norm scores. Effect sizes for univariate time effects on generic HRQoL scores were small to moderate in magnitude.

According to the literature [10,42,43], in our study parents reported larger HRQoL impairments than children themselves. Since the decision to seek treatment depends in large part on parents, only those children whose parents perceive greater impairments may be enrolled for weight management programmes. A further explanation for higher self-reported scores is the ‘response shift’ phenomenon, where children with chronic health conditions adapt to their condition, develop coping strategies and re-adjust assessment standards for well-being [44]. It was also supposed that youth may hesitate to acknowledge negative impacts resulting from their weight [10]. Beyond that it seems also possible, that parents aggrandize problems of their children in the knowledge that overweight is detrimental to health and socially prejudiced. It therefore seems important to study both perspectives [43] and ensure that improvements are also validated with self-reports, which was the case in our study, where self-reported values increased even for dimensions were no significant impairments were evident.

From our results in relation to other studies it can be supposed that the expectable magnitude of positive HRQoL changes during and after weight management training in general varies with the degree of impairment before treatment [7]. Larger increases are to be expected for more impaired HRQoL scores and therefore for proxy-reports, more overweight youth or aspirants for more intensive treatments.

Effects on generic HRQoL were more pronounced when looking at norm scores than on original scores. In general, HRQoL decreases with age during adolescence [28], so that age effects may partially mask time effects. Thus, whenever possible, examination of standardized values seems preferable and has the further advantage of being more easily interpretable.

The observed changes of HRQoL during our study have important implications for future studies on weight management: As improvements continued after end of treatment on most HRQoL dimensions and most scores were not significantly different post-treatment, longer follow-up periods and larger study populations seem necessary to verify psychosocial improvements at least in only moderately overweight children and adolescents. In addition, it may be that in some preceding studies on obese youth more improvements would have been revealed with longer follow-up measurements. This is in line with Tsiros et al. [10], who concluded that changes in psychosocial HRQoL dimensions are less common than changes in physical HRQoL because these changes require more time.

Direct correlations between weight reduction and HRQoL changes were low in our study for the overall sample and mainly significant for weight-specific HRQoL. Other studies confirm these low associations. Studies by Yackobovitch-Gavan and colleagues [18,19], for example, found no significant correlations of weight changes with generic HRQoL during a 12-week intervention. Wille and colleagues [11] found low and non-significant associations with generic and weight-specific HRQoL, while in the study of Patrick et al. [15] associations with generic HRQoL changes were no longer significant when adjusted for baseline scores, whereas correlations with changes in weight-specific HRQoL remained significant. The only significant association found by Fullerton et al. [17] was for changes in physical well-being. Our results concerning associations with improvements in school functioning vary from previous research.

However, unlike other studies we additionally looked at gender-specific associations and found clear gender differences in results, where weight reduction of girls was more favourably correlated with HRQoL changes, while in boys some correlations opposite to the expected direction were found. These gender-specific effects partially averaged out to low correlations for the overall sample. Gender-specific associations were particularly pronounced in terms of long-term changes. None of the previous studies reported associations with long-term changes. Short-term HRQoL changes in our study were often associated with long-term weight changes and pointed to improvements in emotional well-being, self-esteem, school, and weight complaints preceding long-term weight maintenance in girls, while in boys long-term associations were rather unfavourable for generic HRQoL. Possible negative effects as well as gender-specific results should be further monitored, although the gender effects observed in our study may well be sample-specific, for our male subsample was quite small (n = 24). Furthermore, HRQoL scores increased in both boys and girls during and after treatment, so that there is no indication that treatment-induced weight reduction overall showed detrimental effects.

Our results point to HRQoL changes precdicting long-term weight changes in girls rather than vice versa, since on some scales short-term HRQoL changes were associated with long-term weight changes, while no direct correlations were found between long-term changes in HRQoL and long-term weight development. Although no causal effect can be proved by this study, an effect of weight change that occurs later in time on previous HRQoL changes can be ruled out. A possible interpretation, therefore, is that in girls an improved HRQoL, especially in terms of self-esteem and emotional well-being, helped in sustaining a reduced weight, while in boys no such effect was revealed.

In general, weight reduction seems not to be the only critical factor for HRQoL changes during or subsequent to weight management treatment, since it was not consistently associated with HRQoL changes. Further, associations with weight reduction may differ between boys and girls. Because increases in generic HRQoL were not directly related to weight reduction in most cases, improvements may depend primarily on specific content of programme (for example social support or promotion of self-acceptance) more than on diet changes that directly lead to weight reduction, or improvements may not parallel weight changes temporally. This has important implications for practice. While long-term weight reduction may be difficult to achieve for many overweight youth, interventions that lead to HRQoL improvements may increase psychosocial health and well-being even in the absence of weight loss. Hence, they may be an alternative to help those who are not able to achieve a healthy weight in coping with their condition. Future studies should therefore clarify which specific components of the intervention result in HRQoL improvements in girls and boys.

### Strengths and limitations

As far as we know, our study is the first to demonstrate HRQoL-changes during and after treatment in moderately overweight children and adolescents. It has the strengths to track changes on generic and weight-specific HRQoL dimensions over multiple occasions until one year after end of treatment from the perspective of the children themselves as well as from parents’ point of view and relate these changes to weight reduction.

However, there are some limitations to be considered, when interpreting our results. At first, reliability of some HRQoL subscales was unsatisfactory, especially for friends, self-esteem and school; although internal consistency was only slightly lower than in other samples [28,45]. This may have resulted in larger measurement errors and therefore lowered power of statistical tests. Even so, we could demonstrate improvements on these dimensions at least for parent reports, so that this deficiency seems not to have affected our results too much. In future studies, however, more detailed instruments may be preferable in studies on separate HRQoL dimensions. The tendency for social desirable answers in our sample was high. However, we could not find any influence on our results. Because different intervention components were delivered simultaneously, we cannot relate particular components to HRQoL changes. Last but not least, even if the study was designed as RCT, we had no control group to compare our follow-up results to. Given the risk of further weight gain (which is what we observed in untreated controls) we delayed the intervention in the control children for no more than six months. For longer observation periods we had to pool the groups. We therefore cannot draw definite inferences from our analysis about the intervention having caused the observed improvements. Nevertheless, with standardizing HRQoL scores based on age- and gender-specific norms we tried to compensate to some extent for this weakness. Unfortunately, this was not possible in case of weight-specific HRQoL.

## Conclusions

Participation in a six-month outpatient training programme for weight reduction had favourable effects on HRQoL of moderately overweight children and adolescents. Improvements in HRQoL were most notable one year after end of treatment in generic HRQoL and near-term rather in weight-specific HRQoL. These HRQoL improvements were hardly related to weight reduction in the entire sample, but associations were visible in girls. Positive effects on HRQoL were demonstrated for an overall successful treatment, though, and therefore may not hold true for programmes that do not result in weight reduction.

Given that HRQoL improvements continued after treatment, future studies should include longer follow-up periods to document HRQoL development in the long run. An issue that should be further monitored is the potentially negative impact weight reduction may have on some HRQoL-dimensions in boys.

## Abbreviations

HRQoL: Health-related quality of life; zBMI: z-score of body mass index calculated using Coles LMS method.

## Competing interests

The authors declare that they have no competing interests.

## Authors’ contributions

EF participated in the acquisition of data, conceptualized and performed the statistical analyses and drafted the manuscript. PK and TR conceived of the study and its design and helped in interpreting the results. AS, KW, and TR participated in acquisition of the data and were involved in implementing the intervention. All authors critically revised the manuscript and read and approved the final manuscript.
